# Association of white matter hyperintensities with migraine phenotypes and response to treatment

**DOI:** 10.1007/s13760-022-02015-x

**Published:** 2022-07-19

**Authors:** Sherihan Rezk Ahmed, Amr Abdel Monem Mohamed, Haitham Hamdy Salem, Shahinaz Helmy, Ramez Reda Moustafa, Sherien Mohamed Farag Borham

**Affiliations:** 1 Department of Neurology, Faculty of Medicine, Kafr el-Sheikh University, 12 Elgeish street, Kafr el-sheikh, 33511 Egypt; 2 Department of Neurology, Faculty of Medicine, Ain Shams University, Cairo, Egypt

**Keywords:** WMHs, Migraine phenotypes, Migraine treatment response

## Abstract

**Introduction:**

White matter hyperintensities (WMHs) are frequently found in migraineurs. However, their clinical significance and correlation to different migraine phenotypes and treatment responses are not well defined. The study aimed to examine the association of WMHs with migraine clinical patterns and treatment response.

**Aim of work:**

We aimed to evaluate the association between WMHs and migraine phenotypes and explore the relationship of WMHs to treatment response.

**Methods:**

Our cross-sectional study formed of 500 migraineurs who sought treatment in Kafr el-sheik university hospital and underwent (3 T) MRI to evaluate WMHs. Different migraine phenotypes were compared between patients with and without WMHs. According to reduced headache pain intensity and frequency, these patients were divided into treatment responder and non-responder groups.

**Results:**

A total of 145 patients (29%) had WMHs. Patients with WMHs were significantly older, had a longer disease duration, and higher attack frequency. Patients who did not respond to acute and maintenance medications had a higher frequency of WMHs and high WMHs Scheltens score. Migraine with Aura and the presence of vomiting and dizziness were predictors for the development of WMHs.

**Conclusion:**

WMHs are more common in migraine with aura. It is more frequent in migraine associated with vomiting and dizziness. WMHs increased with advancing age and more severe disease burden. Poorer response to acute and prophylactic medications was found in patients with WMHs.

**Supplementary Information:**

The online version contains supplementary material available at 10.1007/s13760-022-02015-x.

## Introduction

Migraine is a primary headache disorder characterized by recurrent moderate-to-severe headaches associated with various autonomic and sensory symptoms [1].

Migraine may present by different phenotypes and clinical patterns such as migraine with aura and without aura, and may have various associated symptoms such as vomiting, nausea, dizziness, photophobia and phonophobia [2].

Large epidemiological studies indicated that migraine could lead to white matter hyperintensities (WMHs) [3].

Some studies reported that WMHs were twice as common in migraine patients as in the general population. In contrast, other authors said that its prevalence is comparable to that of the healthy population [4, 5].

The exact relation between WMHs and the clinical patterns of migraines remains unclear. The population-based CAMERA study suggested the increased risk of WMHs in migraineurs is associated with higher attack frequency. Some authors reported that both disease duration and attack frequency were associated with WMHs in migraineurs [6]. In contrast, others observed an association between WMHs and patients’ age and migraine duration, but not with attack frequency [7].

In this study, we hypothesized that there is a relation between migraine phenotypes and WMHs, and also an association between WMHs and response to treatment.

## Materials and methods

### Sample size

The sample size of this cross-sectional study was based on the study carried out by Yalcin and colleagues 2018 [8]. We used Epi Info STATCALC (Georgia, US, 2018) to calculate the sample size by considering the following assumptions: 95% two-sided confidence level, with a power of 85%, alpha error of 5%, and odds ratio calculated = 1.114. The final maximum sample size, which was taken from the Epi- Info output, was 463. We increased the sample size to 500 patients to account for any dropout cases during the evaluation period.

Our study was conducted between November 2019 and April 2021 in Kafr el-sheik university hospital, where the migraineurs who sought medical treatment were randomly assigned in a 1:1 ratio to be included in the study (615 patients) or not included (617 patients) with the help of a web-based blocked randomization plan.

### Inclusion criteria:

We screened patients aging 10–55 years with migraines according to the International Classification of Headache Disorders 3rd edition [1].

### Exclusion criteria:

Patients with major neurological conditions as (epilepsy, ischemic or hemorrhagic stroke, multiple sclerosis, mitochondrial diseases, brain tumors, patients with essential tremors, were excluded, as well as patients with major systemic diseases as malignancy, collagen diseases, liver diseases, renal diseases and cardiovascular diseases like hypertension (systolic blood pressure more than 130 and/or diastolic blood pressure more than 85 mm/Hg in at least three different occasions[9], diabetes (fasting plasma glucose level > 126 mg/dl and/or a casual plasma glucose > 200 mg/dl and/or HbA1C more than 6.5 [10]. We excluded also patients with valvular and ischemic heart diseases, as well as patients with MRI contraindications, pregnant and lactating patients, and patients received prophylactic treatment for migraine other than topiramate, also we excluded patients with any contraindications to topiramate or ibuprofen.

### Study procedures

We randomly enrolled 615 migraine patients in our study and used a questionnaire to detect their demographic and clinical features (disease duration, attack frequency, and duration, pain intensity assessed by visual analogic scale). Special emphasis was put on clinical phenotype (for example, character of pain, location, associated symptoms, etc.); vascular risk factors (hypertension, diabetes, smoking); history of cerebrovascular events and other conditions (collagen disorders, hepatic disorders, blood diseases, heart, kidneys), and family history.

**Fig. 1:**
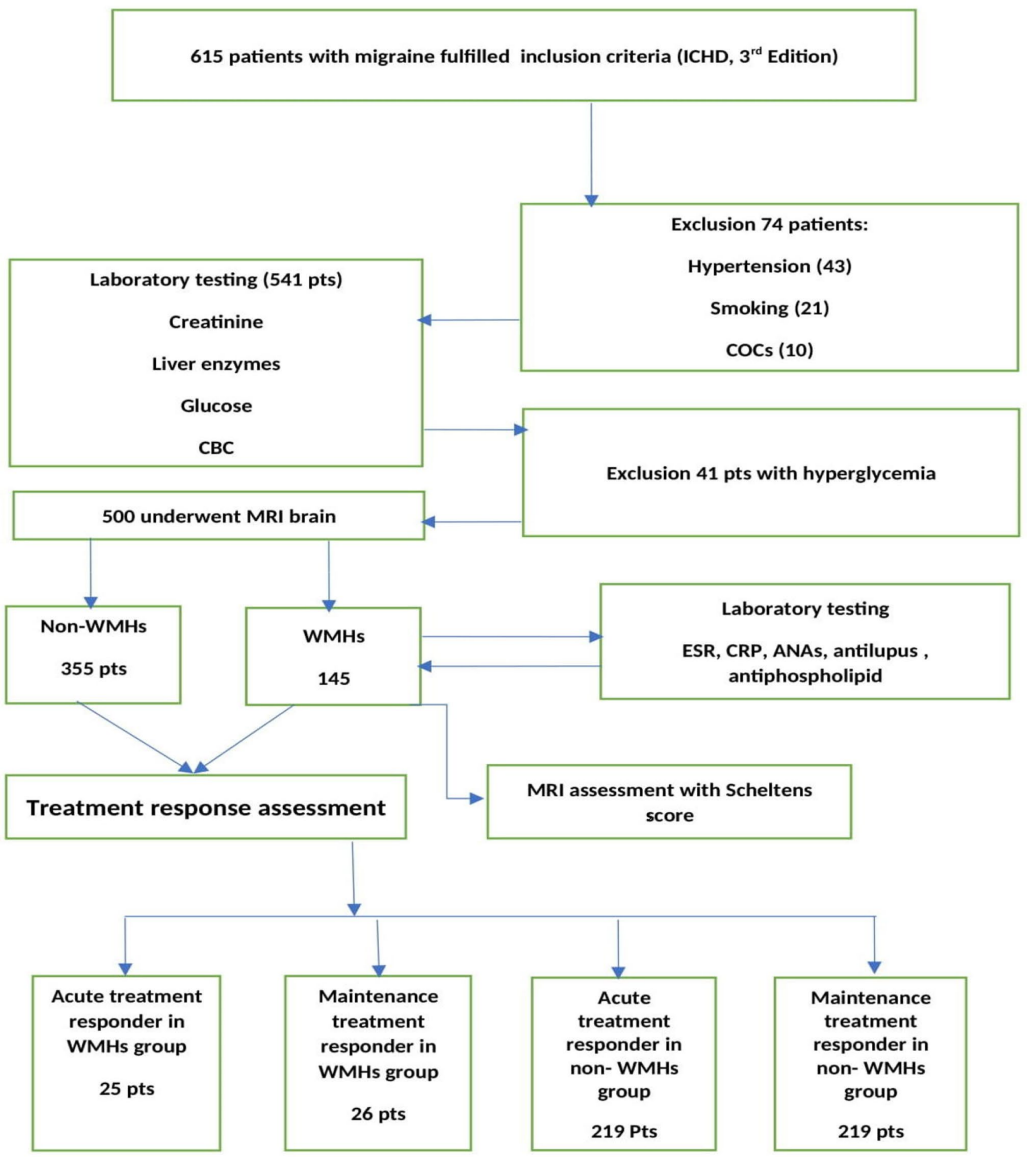
Study flow diagram

All the screened patients underwent clinical neurological and general physical examinations, and migraine history and associated phenotypic features were established, and we measured blood pressure in three different occasions, and performed laboratory tests including (fasting, post-prandial blood sugar and HBA1C, renal functions, liver functions, coagulation profile, complete blood count), and 43 patients were excluded due to hypertension, twenty one, ten, forty one patients were ruled out due to being smokers, taking oral contraceptives, and having high blood glucose level respectively, as shown in **(**Fig. 1).

We excluded the Patients with major neurological conditions as (epilepsy, ischemic or hemorrhagic stroke, multiple sclerosis, mitochondrial diseases, brain tumors, patients with essential tremors based on history.

**Fig. 2:**
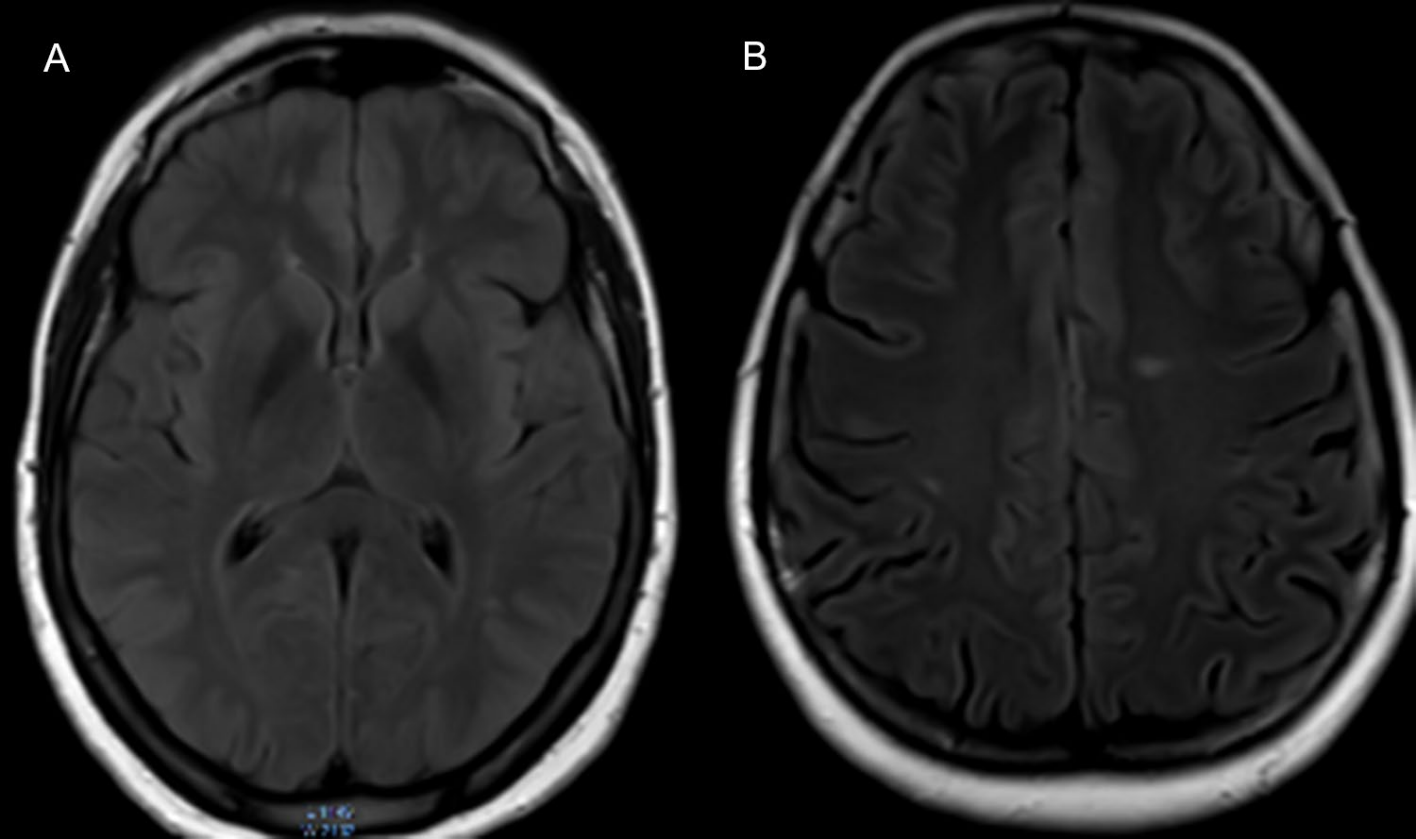
Representative axial FLAIR images of WMHs: (A) Normal brain structures without white matter hyperintensity. (B) punctate hyperintense lesions in the both parietal lobes

Based on history, we excluded also patients with valvular and ischemic heart diseases, as well as patients with MRI contraindications, pregnant and lactating patients, and patients received prophylactic treatment for migraine other than topiramate, also we excluded patients with any contraindications to topiramate or ibuprofen.

All the remaining 500 patients who fulfilled inclusion criteria underwent routine laboratory tests (fasting, post-prandial blood sugar and HBA1C, renal functions, liver functions, coagulation profile, complete blood count), ECG monitoring, transthoracic echocardiography, MRI brain, and we excluded patients with any abnormality retrogradely.

Patients who had WMH were also tested for levels of lupus anticoagulant, CRP, ESR, antiphospholipid antibodies and antinuclear factor, and we excluded patients with any abnormality retrogradely.

All the patients underwent brain MRI (T1W, T2W, FLAIR) on a Philips 3 T Achieva scanner (Philips, The Netherlands) using an eight-channel head RF array coil: 3D-T1-weighted gradient echo scan (TR = 28 ms, TE = 4 ms, 60 axial slices acquired at 3 mm slice thickness, in-plane voxel size = 1 × 1 mm^2^, flip angle = 27, FOV = 250 × 188 × 180 mm^3^). T2-weighted (TR = 6,100 ms, TE = 80 ms, 60 axial slices acquired at 3 mm slice thickness, in-plane voxel size = 1 × 1 mm^2^, FOV = 250 × 188 × 180 mm^3^, ETL = 8), and a FLAIR (TR = 9000 ms, TE = 80 ms, TI = 2,500 ms, 60 axial slices acquired at 3 mm slice thickness, in-plane voxel size = 1 × 1 mm^2^, FOV = 250 × 188 × 180 mm^3^, ETL = 12).

The pattern of WMHs was assessed by two neurologists and one radiologist using the Scheltens visual rating scale. The physicians who assessed the imaging were blinded to the participant’s migraine status, each other’s ratings, and the nature of the study. Disagreement was settled by reaching a consensus decision. WMHs were graded in the following locations: frontal lobes, temporal lobes, parietal lobes, occipital lobes and as follows: 0 (no lesions), 1 (hyperintensity < 3 mm and n ≤ 5), 2 (hyperintensity < 3 mm and n ≥ 6), 3 (hyperintensity 4– 10 mm and n ≤ 5), 4 (hyperintensity 4–10 mm and n ≥ 6), 5 (hyperintensity ≥ 11 mm and n ≥ 1), and 6 (confluent). The sum of scores from each location was considered as the final score. According to WMH, migraine patients were divided into two groups: non-WMH group (WMHs score = 0) and WMH group (WMHs score ≥ 1) [11].

As regards assessment of treatment response, all patients in our study received Ibuprofen (200–400 mg) [12] as a treatment to acute attack and topiramate (25–200 mg) as a prophylactic treatment for at least 2 months [13].

We considered a patient a “responder to acute treatment” when they achieved pain freedom within 2 h in ≥ 4 of 5 attacks while insufficient responders achieved pain freedom in ≤ 3 of 5 attacks [14]. As for a response to preventative treatment, we considered a patient a responder to “preventative treatment” when there was a ≥ 50% reduction in the monthly headache days frequency compared to the baseline frequency [15].

### Primary outcome

Association between WMHs and different migraine phenotypes.

### Secondary outcome

Association between WMHs and migraine treatment response.

### Statistical analysis

This is the primary statistical analysis of our data was done through IBM SPSS software package version 20 (Armonk, NY: IBM Corp). The primary and secondary outcomes were subjected to separate two-tailed statistical analysis.

Continuous data were analyzed through median and interquartile range [IQR], the Shapiro–Wilk test was used, categorical data were represented in numbers and percentage.

Pearson’s chi-square was used to correlate categorical data. In contrast, the Mann–Whitney *U* test was used for the abnormally distributed numerical data. In our study, there were no missing data.

## Results

Five hundred migraine patients completed the study (230 males and 270 females). The age ranged between 10 and 55 years with a median value of 36 years and interquartile range (IQR) from 27 to 48 years. The age group 20–40 years comprised 48.8% of patients. Photophobia was present in 89% of patients, 88% had phonophobia, 80% had nausea, 71% had dizziness. In comparison, only 39.6% had vomiting. We found that 150 patients had migraine with aura, and that 235 satisfied the criteria for chronic migraine. White matter hyperintensities were found in 145 patients on MRI (Table 1**)**, (Fig. 2).

**Table 1:** Association between demographic data and the presence of WMHs

Demographic data	All patients (*n* = 500)	Non-WMH group (*n* = 355)	WMH group (*n* = 145)	*P* value
Age (years)				
10–20, no. (percentage)	58 (11.6%)	49 (13.8%)	9 (6.2%)	< 0.001
21–30, no. (percentage)	124 (24.8%)	103(29.0%)	21 (14.5%)	
31–40, no. (percentage)	125 (25.0%)	97 (27.3%)	29 (20.0%)	
41–50, no. (percentage)	105 (21.0%)	67 (18.9%)	36 (24.8%)	
51–55, no. (percentage)	88 (17.6%)	39 (11.0%)	50 (34.5%)	
Median (IQR)	36.0 (27.0–48.0)	23(26–44)	45(33–54)	
Sex				
Female, no. (percentage)	230 (46.0%)	191 (53.8%)	79 (54.5%)	0.892
Male, no. (percentage)	270 (54.0%)	146 (46.2%)	66 (45.5%)	
Migraine phenotypes				
Nausea, no. (percentage)	400 (80.0%)	290 (81.7%)	110 (75.9%)	0.191
Vomiting, no. (percentage)	198 (39.6%)	130 (36.6%)	68 (46.9%)	0.033
Photophobia, no. (percentage)	445 (89.0%)	315 (88.7%)	130 (89.7%)	0.791
Phonophobia, no. (percentage)	440 (88.0%)	311 (87.6%)	129 (88.9%)	0.812
Dizziness, no. (percentage)	355 (71.0%)	251 (70.8%)	104 (71.7%)	0.872
Aura, no. (percentage)	150 (30.0%)	59 (16.6%)	91 (62.8%)	< 0.001
Migraine character				
Throbbing, no. (percentage)	300 (60.0%)	211 (59.4%)	89 (61.4%)	0.591
Dull, no. (percentage)	47 (9.4%)	35 (9.9%)	12 (8.3%)	0.762
Stabbing, no. (percentage)	67 (13.4%)	43 (12.1%)	24 (16.6%)	0.131
Pressing, no. (percentage)	86 (17.2%)	66 (18.6%)	20(13.8%)	0.472
Migraine localization				
Unilateral, no. (percentage)	186 (37.2%)	130 (36.6%)	56 (38.6%)	0.572
Bifrontal, no. (percentage)	86 (17.2%)	60 (16.9%)	26 (17.9%)	0.651
Generalized, no. (percentage)	98 (19.6%)	68 (19.2%)	30 (20.7%)	0.572
Occipital, no. (percentage)	77 (15.4%)	55 (15.5%)	22 (15.2%)	0.911
Others, no. (percentage)	53 (10.6%)	42 (11.8%)	11 (7.6%)	0.643
Migraine chronicity				
Episodic migraine, no. (percentage)	265 (53.0%)	194 (54.6%)	71 (49%)	0.751
Chronic migraine, no. (percentage)	235 (47.0%)	161 (55.4%)	74 (51%)	0.672

Significant *P* value < 0.05

When we compared non-WMHs group and WMHs group regarding migraine phenotypes, we found that patients in the WMHs group were significantly older than those in the non-WMHs group (*P* < 0.001) as 59.3% of patients lie within the age group (41–55). Patients in the WMHs group also more frequently had migraine with aura (*P* < 0.001) and more frequently had associated vomiting with their migraines (*P* = 0.03) (Table 1).

We also found that the WMHs group had a statistically significant longer headache duration, longer interval between the patient’s first-ever migraine attack and the time when the patient had the brain MRI, more frequent attacks, longer attack duration, higher pain intensity when compared with the non-WMHs group (*P* < 0.001) Table 2.

**Table 2:** Association between headache characters and the presence of WMHs

Headache character	Non-WMH group (*n* = 355)	WMH group (*n* = 145)	*U*	*P*
Disease duration (month, median, IQR)	60.0 (36.0–120.0)	108.0 (72.0–120.0)	17,454.3	0.001
Interval between the first-ever migraine to MRI enrollment (month, median, IQR)	56.0 (36.0–114.0)	100.0 (60.0–110.0)	17,922.0	0.01
Attack frequency (day/month, median, IQR)	3.0 (3.0–4.0)	5.0 (5.0–7.0)	8339.5	< 0.001
Attack duration (hour median, IQR)	5.0 (4.0–5.0)	5.0 (5.0–6.0)	14,761.3	< 0.001
VAS (median, IQR)	5.0 (5.0–5.0)	6.0 (5.0–7.0)	10,094.5	< 0.001

Significant *P* value < 0.05

Also, we found that migraine with vomiting was associated with a significantly higher number of WMHs lesions with (*P* = 0.01). Migraine with aura was associated with a significantly higher Scheltens score (*P* = 0.007). The other migraine phenotypes had no difference regarding the number of WMHs or Scheltens score, as shown in Table 3.

**Table 3:** Association between migraine phenotype and WMHs number and Scheltens score

Phenotype	State	Patients number	Number of WMHs (median, IQR)	*U*	*P* value	Scheltens Scale score (median, IQR)	*U*	*P* value
Nausea	Present	400	2 (1–3)	1354	0.090	1.5 (1–2)	1490	0.580
	Absent	100	2 (2–3)			2 (1–3)		
Vomiting	Present	198	4 (1–4)	815.6	0.010	1 (1–3)	2342	0.231
	Absent	302	2 (2–3)			2 (1–2)		
Photophobia	Present	445	2(2–4)	2147	0.120	1 (1–2)	806.5	0.242
	Absent	55	2(2–4)			2 (2–3)		
Phonophobia	Present	440	2(2–4)	2471	0.411	1 (1–2)	1041.2	0.311
	Absent	60	2(2–4)			2 (2–3)		
Dizziness	Present	355	2 (2–3)	1456.2	0.710	2 (1–2)	1632.2	0.281
	Absent	145	2 (2–3)			1 (1–2)		
Aura	Present	150	4 (1–5)	1125.2	0.030	2 (1–3)	1945.3	0.007
	Absent	350	2 (2–4)			1 (1–2)		
Migraine chronicity	Episodic	265	2 (2–3)	2034.2	0.644	3(1–4)	1842	0.123
	Chronic	235	2 (2–3)			2(1–4)		
Throbbing pain	present	300	3 (1–3)	1243	0.092	2 (1–3)	1723	0.273
	absent	200	3 (2–3)			2 (2–3)		
Dull aching	present	47	2 (1–3)	1425	0.571	1.5 (1–2)	1651	0.612
	absent	453	2 (2–3)			2 (1–3)		
Stabbing pain	present	67	2 (1–2)	2417	0.421	2 (1–3)	1754	0.121
	absent	433	1 (1–2)			2 (2–3)		
Pressing pain	present	86	2(2–4)	1736	0.531	1 (1–2)	2031	0.412
	absent	414	2(2–4)			2 (2–3)		
unilateral	present	186	2 (1–3)	1124	0.712	2 (2–3)	921.1	0.811
	absent	315	2 (2–3)			2 (2–3)		
bifrontal	present	86	2 (1–3)	1563	0.391	3 (1–3)	1239	0.081
	absent	414	2 (2–3)			3 (2–3)		
generalized	present	98	2 (1–2)	2117	0.323	2 (1–3)	1951	0.512
	absent	402	1 (1–2)			2 (2–3)		
occipital	present	77	2(2–4)	1236	0.271	1 (1–2)	1354	0.081
	absent	423	2(2–4)			2 (2–3)		

Significant *P* value < 0.05

When we compared the response to acute treatment, we found that acute treatment non-responders more frequently had WMHs than treatment responders (*P* < 0.001). They had larger WMH lesions with a mean diameter of 3.5 ± 1.9 mm compared with 3.2 ± 1.8 mm in acute treatment responders (P < 0.001). Also acute treatment non-responders had higher Scheltens score and WMHs number (*P* < 0.001 for both). Similarly, we found that preventative treatment non-responders more frequently had WMHs (P˂0.001), and had larger WMH lesions with a mean diameter of 3.42 ± 1.9 mm compared with 3.24 ± 1.8 mm in preventative treatment responders (*P* < 0.001). Also preventative treatment non-responders had higher Scheltens score and WMHs number (*P* < 0.001 for both) as shown in Table 4.

**Table 4:** Association of WMHs with acute and maintenance treatment response

WMHs characters	responders to acute treatment (244)	Non-responders to acute treatment (256)	*P* value	responders to maintenance treatment (245)	Non-responders to maintenance treatment (255)	*P* value
Presence of WMHs, percentage	25 (10.2%)	120 (46.9%)	< 0.001	26 (10.6%)	119 (46.7%)	< 0.001
Maximum lesion diameter in millimeters						
≤ 3 mm	24	110	< 0.001	25	109	< 0.001
4–10 mm	1	6		1	6	
≥ 11 mm	0	2		0	2	
Confluent	0	2		0	2	
Median ( IQR)	3 (3–3)	3 (3–3)	< 0.001	3 (3–3)	3 (3–3)	< 0.001
Scheltens score, median, IQR	1 (1–1)	2 (1–3)	< 0.001	1 (1–1)	2 (1–3)	< 0.001
Number of WMHs, median, IQR	1 (1–2)	4 (2–5)	< 0.001	1 (1–2)	4 (2–5)	< 0.001

Significant *P* value < 0.05

When we employed multiple linear regression analysis using multiple predictor variables (migraine phenotypes: nausea, vomiting, photophobia, phonophobia, and dizziness) to predict a single outcome (development of WMHs in MRI), we found that neither nausea, photophobia nor phonophobia had a statistical significant correlation with the development of WMHs. Also, the presence of vomiting and dizziness had a statistically significant positive association with the development of WMHs, as shown in Table 5.

**Table 5:** Multiple linear regression analysis of the development of WMH among migraineurs

Migraine phenotype	WMHs in MRI	
	rs	*P* value
Nausea	0.04	0.321
Vomiting	0.24	0.010
Photophobia	0.01	0.762
Phonophobia	0.02	0.721
Dizziness	0.12	0.050

Significant *P* value < 0.05

*rs* regression square

## Discussion

White matter hyperintensities (WMHs) are frequently found in migraineurs, yet their significance and correlation to migraine features remains a matter of debate and conflicting results [11]. In our study, we aimed to examine the association between WMHs and migraine phenotypes and explore the relationship of WMHs to treatment response.

We found that 29% of all migraine patients had WMHs (26.8% of episodic and 31.5% of chronic migraine patients). The incidence of WMHs increased with age, longer disease duration, longer attack duration, and higher attack frequency. They were also much more frequently found in patients who had migraine with aura (62% of patients). No particular clinical features were associated with WMH except for vomiting and dizziness during attacks.

Our findings show a lower prevalence of WMH than reported by Seneviratne et al. who found that 43% of migraine patients had WMHs and Xie et al. who found that 35% of their patients had WMHs. Conversely, other studies such as that of Markus et al. have found that only 11% of their migraine patients had WMHs. The variation could be explained by many methodological differences but also by the fact that the mean age of our patients was 27 years, whereas in Xie et al. was 34 years, in Seneviratne et al. was 45 years and in Markus et al. was 11 years. It is well known that aging is a significant risk factor for the development of WMHs [11, 16–18]. In the present study, we indeed found that patients with WMHs were significantly older than those without WMHs and that they had a significantly longer disease duration, longer attack duration, and higher attack frequency. These results agree in part with Trauninger et al. who found that patients with WMHs were significantly older than those without WMHs. They had a longer disease duration and higher attack frequency, but there were no significant differences related to attack duration. Similarly, Xie et al. found patients with WMHs were significantly older and had longer disease duration than those without WMHs, but they did not find an effect of attack duration and frequency [6, 11].

These associations between severity of WMH and migraine patterns were not found in the study by Dobrynina et al. [19].

The relationship of disease duration, attack frequency and attack duration to WMH may be explained by the probable tissue damage occurring as a result of several pathologic processes including intracerebral hemodynamic changes, local inflammatory responses, excessive neuronal activation, and excitotoxicity, all of which may lead to tissue damage [20].

We found that WMHs were significantly higher in number in patients with migraine with aura. Our findings agree with Kurth et al., 2011; Bashir et al., 2013 and Kruit et al., 2010 who showed that migraineurs with aura were at increased risk of WMHs. This is postulated to be related to fluctuations in cerebral blood flow associated with hyper-perfusion or hypoperfusion, which is modulated by cortical spreading depression from recurrent aura attacks that affect microvascular hemodynamics leading to ischemic injury [21–24].

On the other hand, our findings contradict the results of Xie et al., Seneviratne et al., Gaist et al. and Dobrynina et al. who found that the presence of aura did not affect the development of WMHs [11, 16, 19, 25]. Our results are difficult to reconcile with some population-based studies, particularly the study by Gaist et al. looking at migraine with aura patients. Clinic-based studies are potentially biased by the preponderance of severe cases and by patients who have multiple comorbidities. We thus took care to exclude patients who have comorbidities to avoid this bias in the results. Also, the sample was balanced between patients who had episodic and chronic migraine. It also had a reasonable percentage of migraine with aura patients (~ 30%) to allow us to draw sound conclusions about this phenotype [25].

The other point that this study addressed was the relationship of WMH to response to treatment. We found that 89.8% of patients who responded to acute and preventive migraine treatment did not have WMHs, and reciprocally, the non-responder group had more frequent WMHs, and a higher Scheltens score than the responder group. This result is in agreement with the findings of Xie et al. who showed that 87% of the patients who responded to migraine treatment did not have WMHs, and with the findings of Alkhaffaf et al. who showed that 91% of improved patients did not have WMHs [11, 26].

We found that migraine associated with vomiting had significantly less frequent response to acute and preventative migraine treatment. This result agrees with Lombard et al. who stated that vomiting was significantly higher in insufficient responders compared with responders [14].

We found that aura was significantly associated with increased incidence of WMHs development. The migraine visual aura is produced by the effect of cortical spreading depression (CSD) [27], which may cause inflammation and release nociceptive substances, vasodilation, endothelial dysfunction, and activation of nociceptive afferents [28].

This study was cross-sectional and thus it could not draw conclusions on whether or not WMHs and migraine are causally related. Similarly, the absence of longitudinal data makes any conclusions about treatment response purely historical. Yet, since there is a strong suggestion that WMHs affect treatment response, this warrants investigation in future longitudinal studies.

The lack of a control group of healthy non-migraine subjects is a potential limitation, but we did not intend to compare migraine to the general population. Instead, our study was designed to assess as accurately as possible the association of WMH with migraine and its features.

## Conclusion

Our study concludes that WMHs are common in migraine and more so in migraine with aura. Migraine patients who experience vomiting and dizziness are more likely to have MRI WHMs, as well as patients with higher headache frequency, longer disease duration and advancing age. Furthermore, the presence of MRI WMHs is associated with poor response to acute and preventative headache medications.

## Supplementary Information

Below is the link to the electronic supplementary material.

Supplementary file1 (DOCX 13 KB)

Supplementary file2 (PDF 37 KB)

## Data Availability

The data of the current study are not publicly available due to the ethical regulations of our university, but are available from the first author (Sherihan Rezk Ahmed) upon reasonable request.
