# Supplementary material for: Association of white matter hyperintensities with migraine phenotypes and response to treatment
Source: Acta Neurol Belg. 2022 Jul 19;123(5):1725–33. doi: 10.1007/s13760-022-02015-x (PMC10505107; doi:10.1007/s13760-022-02015-x)
Supplement: Supplementary file 1 — Supplementary file1 (DOCX 13 KB) [file 13760_2022_2015_MOESM1_ESM.docx]

| **Type of hyper intensity** | ***score*** | | | | | | |
| --- | --- | --- | --- | --- | --- | --- | --- |
| **periventricular Hyperintensities** |  | | | | | | |
| occipital | **0** | **1** | **2** |  |  |  |  |
| frontal | **0** | **1** | **2** |  |  |  |  |
| Lateral ventricle | **0** | **1** | **2** |  |  |  |  |
| **periventricular Hyperintensities total score** |  | | | | | | |
| **White Matter Hyperintensities** |  | | | | | | |
| frontal | **0** | **1** | **2** | **3** | **4** | **5** | **6** |
| parietal | **0** | **1** | **2** | **3** | **4** | **5** | **6** |
| Temporal | **0** | **1** | **2** | **3** | **4** | **5** | **6** |
| occipital | **0** | **1** | **2** | **3** | **4** | **5** | **6** |
| **White Matter Hyperintensities total score** |  | | | | | | |
| **Scheltens score** |  |  |  |  |  |  |  |

***Modified Scheltens et al (Minimum, 0; Maximum, 30)***

***Periventricular Hyperintensities (Minimum, 0; Maximum 6)***

Scoring is as follows: caps, occipital 0/1/2 and frontal 0/1/2; bands, lateral ventricles 0/1/2 (0=absent, 1=≤5 mm, 2=≥6 mm and ≤10 mm).

***White Matter Hyperintensities (Minimum, 0; Maximum, 24)***

Scoring is as follows: frontal 0/1/2/3/4/5/6, parietal 0/1/2/3/4/5/6, occipital 0/1/2/3/4/5/6, temporal 0/1/2/3/4/5/6 (0=no abnormalities, 1=≤3 mm, n≤5; 2=≤3 mm, n≤6; 3=4 to 10 mm, n≤5; 4=4 to 10 mm, n≥6; 5=≥11 mm, n≥1; 6=confluent).
