# Supplementary material for: Association of white matter hyperintensities with migraine phenotypes and response to treatment
Source: Acta Neurol Belg. 2022 Jul 19;123(5):1725–33. doi: 10.1007/s13760-022-02015-x (PMC10505107; doi:10.1007/s13760-022-02015-x)

Figures: Tools Commonly Used to Rate Pain

Visual Analogue Scale

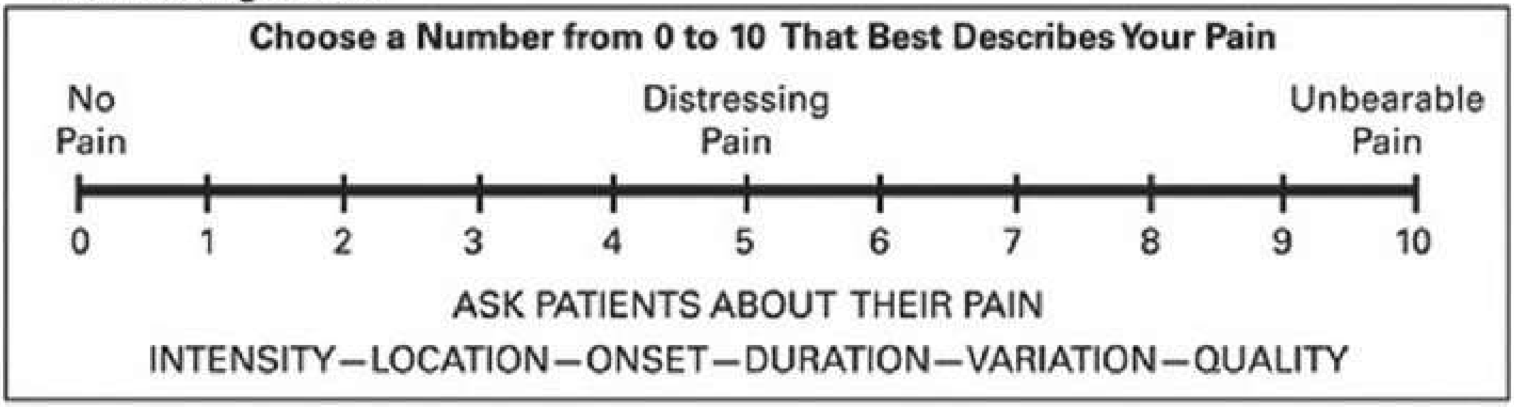

"Faces" Pain Rating Scale

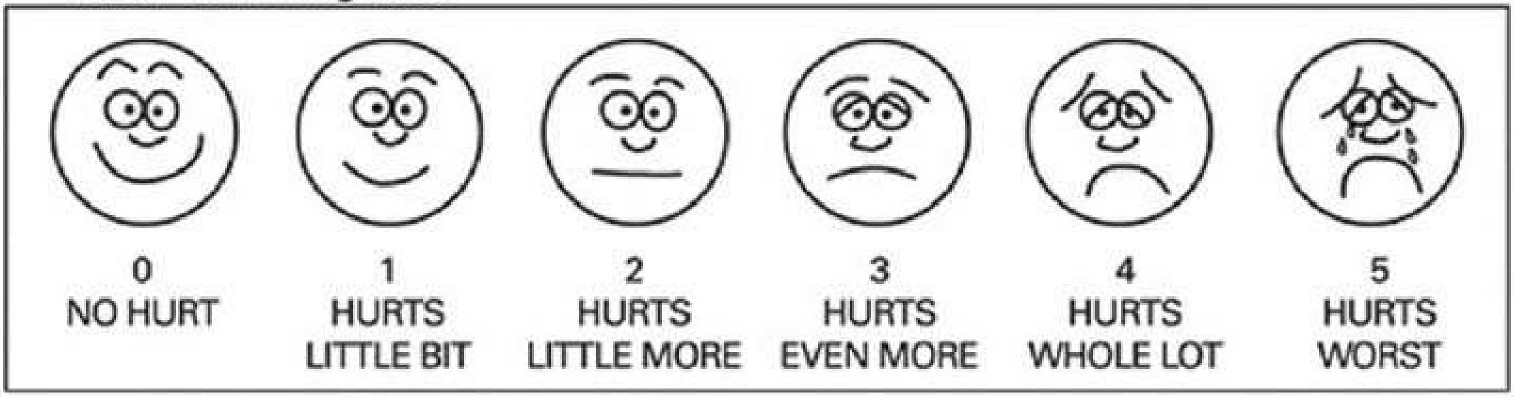

Supplement: Supplementary file 2 — Supplementary file2 (PDF 37 KB) [file 13760_2022_2015_MOESM2_ESM.pdf]
